# A reduced scapulo-humeral angle contributes to the development of scapular notching in reverse total shoulder arthroplasty

**DOI:** 10.1007/s00264-024-06343-w

**Published:** 2024-10-08

**Authors:** Carlo Minoli, Martino Travi, Riccardo Compagnoni, Simone Radaelli, Alessandra Menon, Daniele Marcolli, Alberto Tassi, Pietro S. Randelli

**Affiliations:** 1 U.O.C Week Surgery, ASST Centro Specialistico Ortopedico Traumatologico Gaetano Pini-CTO, Piazza Cardinal Ferrari 1, 20122 Milan, Italy; 2 Università degli Studi di Milano, Via Festa del Perdono 7, 20122 Milano, Italy; 3 1° Clinica Ortopedica, ASST Centro Specialistico Ortopedico Traumatologico Gaetano Pini-CTO, Piazza Cardinal Ferrari 1, 20122 Milan, Italy; 4 Department of Biomedical, Surgical and Dental Sciences, Università degli Studi di Milano, Via della Commenda,10. 20122, Milan, Italy; 5 Laboratory of Applied Biomechanics, Department of Biomedical Sciences for Health, Università degli Studi di Milano, Via Mangiagalli 31, 20133 Milan, Italy; 6 Dipartimento di Scienze Cliniche e di Comunità, Università degli studi di Milano, Milan, Italy; 7 Research Center for Adult and Pediatric Rheumatic Diseases (RECAP-RD), Department of Biomedical Sciences for Health, Università degli Studi di Milano, Via Mangiagalli 31, 20133 Milan, Italy

**Keywords:** Scapular notching, Reverse total shoulder arthroplasty, Scapulo humeral angle, Rotator cuff arthropathy, Shoulder arthroplasty

## Abstract

**Purpose:**

Scapular Notching (SN) is one of the most common postoperative complications for a patient after Reverse Total Shoulder Arthroplasty (RTSA). Despite employing various strategies to mitigate SN risk, the overall incidence remains far from zero. This article introduces a new risk factor, the scapulo-humeral angle (SHA), as a key element influencing the risk for SN.

**Methods:**

A retrospective analysis was conducted on all patients who underwent RTSA for rotator cuff arthropathy at the study centre. The preoperative SHA was measured, and the presence of SN was investigated and graded using the Nerot classification at the latest follow-up.

**Results:**

42 patients were included. 12 presented SN (incidence 28.5%). A statistically significant Pearson coefficient correlation between pre-operative SHA and the incidence of SN was observed (*r*= -0.6954; 95% C.I. -0,8250 to -0,4963; *p* < 0.0001). A statistically significant Pearson coefficient correlation was also found between the degree of SN and the pre-operative SHA (*r*= -0,7045; 95% C.I. -0,8306 to -0,5096; P value (two-tailed) < 0,0001, alpha 0.05).

**Conclusions:**

The primary finding is a statistically significant correlation between a reduced preoperative SHA and an increased incidence of postoperative SN. The secondary finding is that a smaller preoperative SHA is associated with a more severe degree of SN A SHA cut-off of 50° distinguished patients at high risk of SN from those at low risk. All patients with an SHA below 50° developed SN (10/10), whereas only 6.25% of patients with an SHA exceeding 50° experienced SN (2/32).

## Introduction

Reverse Total Shoulder Arthroplasty (RTSA) has gained considerable popularity in recent decades for its success in treating irreparable Rotator Cuff (RC) tears and shoulder arthropathy.[1; 3; 19] Despite being recognized as a safe and effective procedure, RTSA is not without its risks and complications. [4; 7] Among these, Scapular Notching (SN) stands out as one of the most common and potentially severe postoperative issues [6].

SN is characterized by an erosive lesion on the inferior scapular neck, resulting from the impingement of the humeral implant with the scapular neck. A significant number of studies in literature has explored this complication, delving into its causes and potential surgical solutions.[5; 15].

Various studies indicate that specific factors can influence the incidence of SN. These include the utilization of an eccentric glenoid component, an increased glenosphere tilt, a strategically positioned baseplate (inferiorly, laterally, and posteriorly), a lower neck-shaft angle of the humeral component, a deltopectoral approach and an extended scapula neck distance. Despite these insights, SN remains a prevalent complication of RTSA.[2; 6; 8; 9; 12; 14; 16].

Even with a comprehensive understanding and consideration of these factors, SN persists as a common complication, suggesting the existence of additional, yet-to-be-identified risk factors that warrant further investigation.

**Fig. 1:**
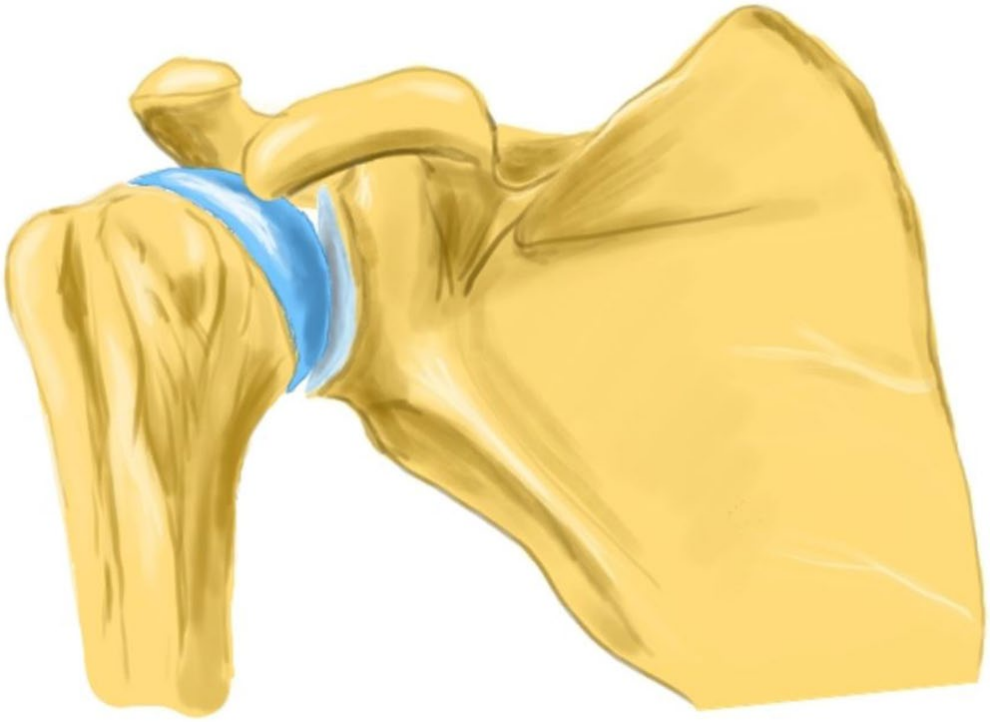
A normal shoulder joint seen in antero-posterior view. [Made with KRITA 4.4.8]

**Fig. 2:**
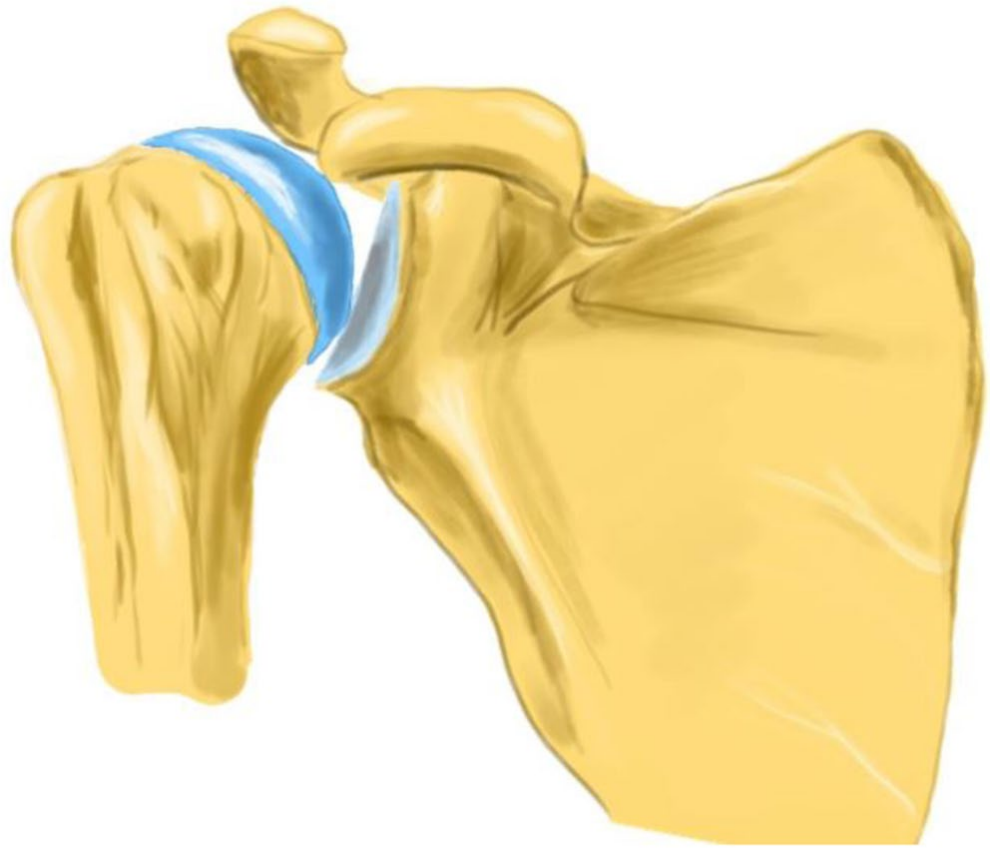
A antero-posterior view of a shoulder with a Rotator Cuff Arthropathy (RCA). *The humeral head goes up against the acromion and the scapula has an upward rotation to compensate for the lack of supraspinatus strength in the abduction-elevation movement*. [Made with KRITA 4.4.8]

This study was motivated by the observation that individuals with Rotator Cuff Arthropathy (RCA) typically seek the expertise of an orthopaedic surgeon after enduring years of shoulder dysfunction and pain. The biomechanics of a shoulder with an irreparable RC tear differ significantly from those of a normal shoulder. In these patients, the initial synergy between the supraspinatus and deltoid muscles, responsible for abduction strength, is compromised. Consequently, the deltoid must bear the entire burden of abduction, working at a disadvantageous leverage during the initial degrees of movement. To compensate, a scapular upward rotation occurs, enhancing the leverage of the deltoid in the early stages of shoulder abduction (see Figs. 1 and 2).

**Fig. 3:**
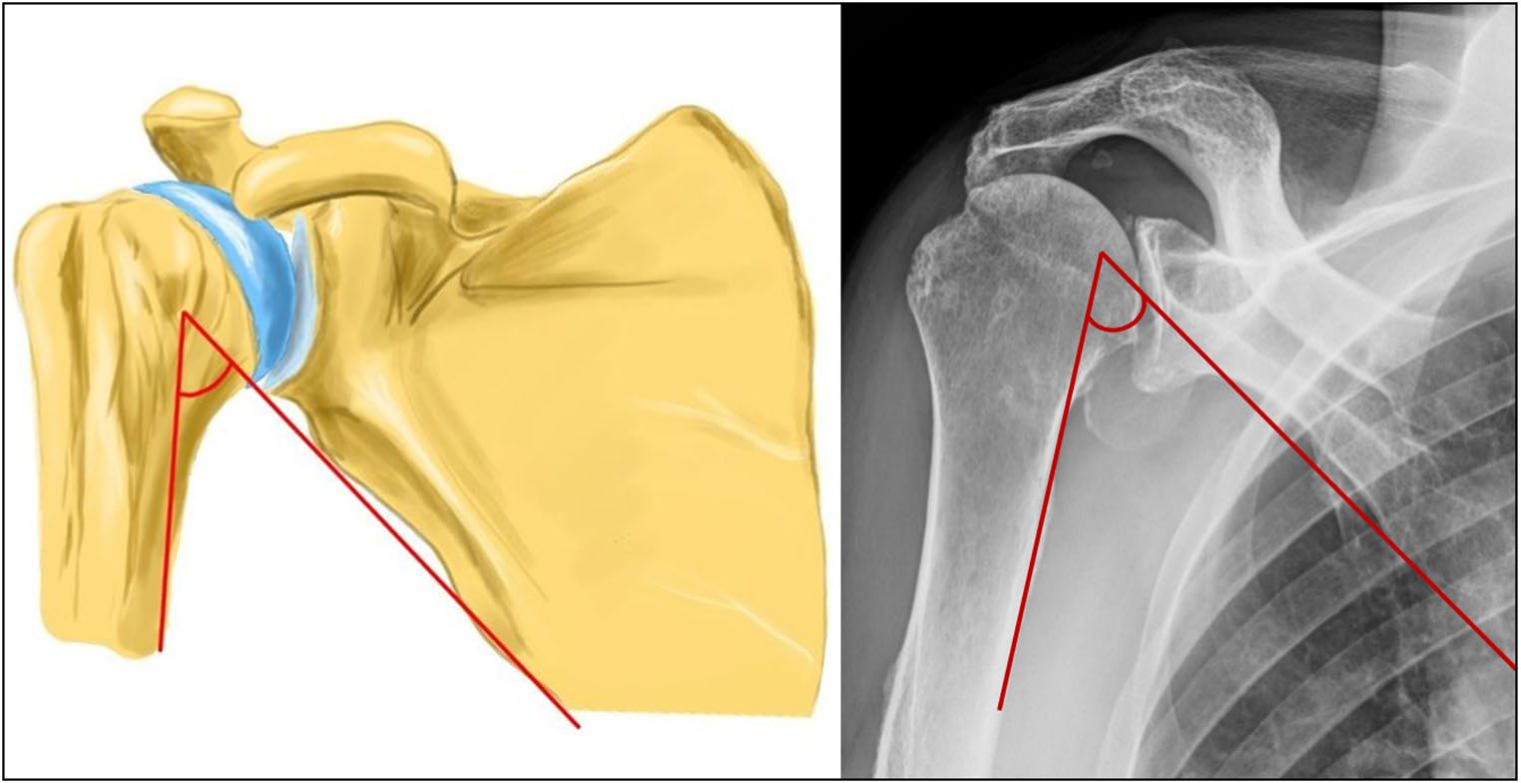
Scapulo-humeral angle in a normal shoulder (defined as the angle between the inferior border of the scapula and the humeral shaft). [Made with KRITA 4.4.8]

**Fig. 4:**
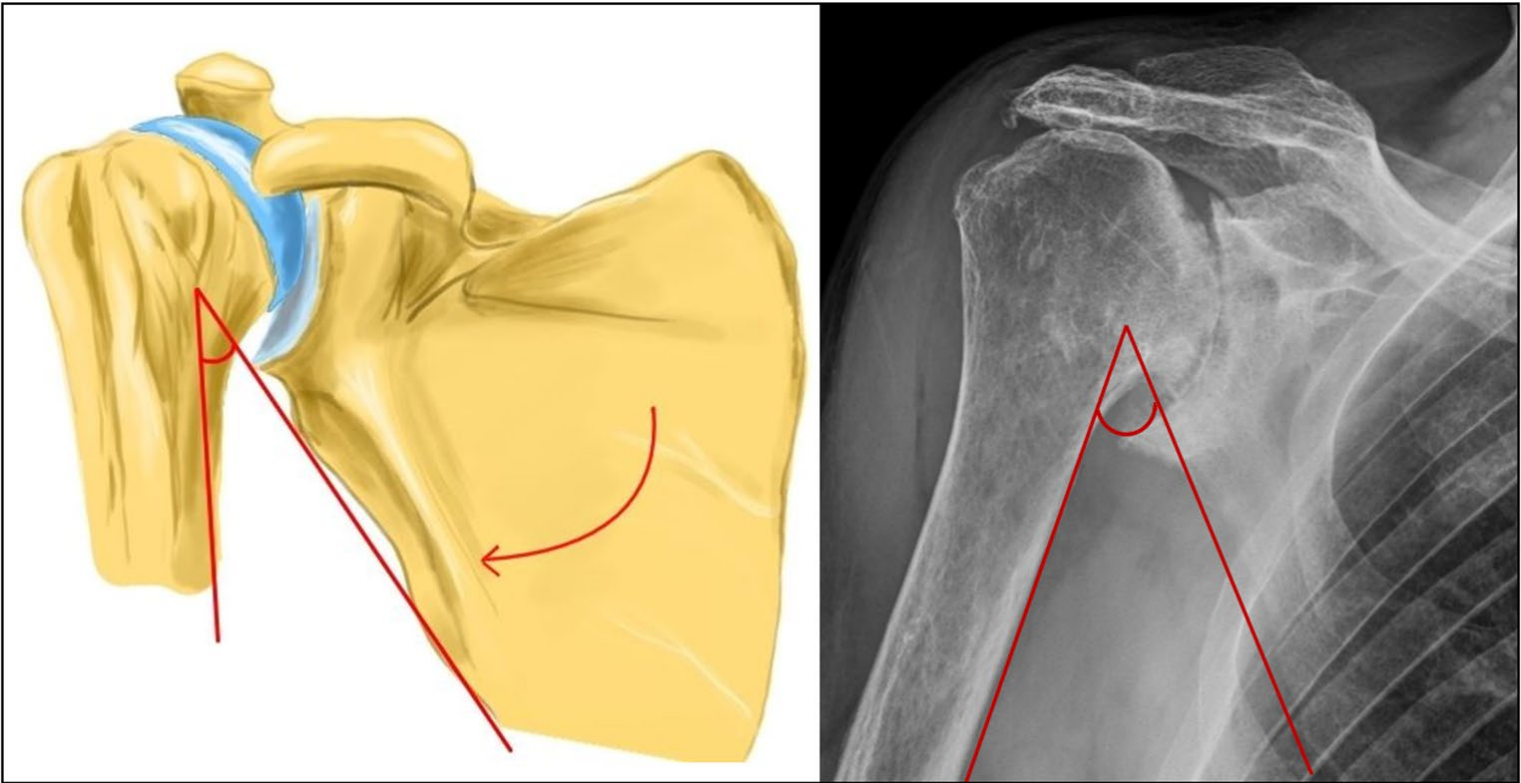
Scapulo-humeral angle (SHA) in a shoulder with Rotator Cuff Arthropathy (RCA). The SHA is reduced due to a compensatory upward rotation of the scapula (arrow). [Made with KRITA 4.4.8]

Our hypothesis suggests that this compensatory movement may progress into chronic scapula-thoracic dyskinesia, leading to a permanent reduction in the angle between the inferior border of the scapula and the humeral shaft axis. For the sake of simplicity, we have termed this angle the Scapulo-Humeral Angle (SHA) (refer to Figs. 3 and 4).

**Fig. 5:**
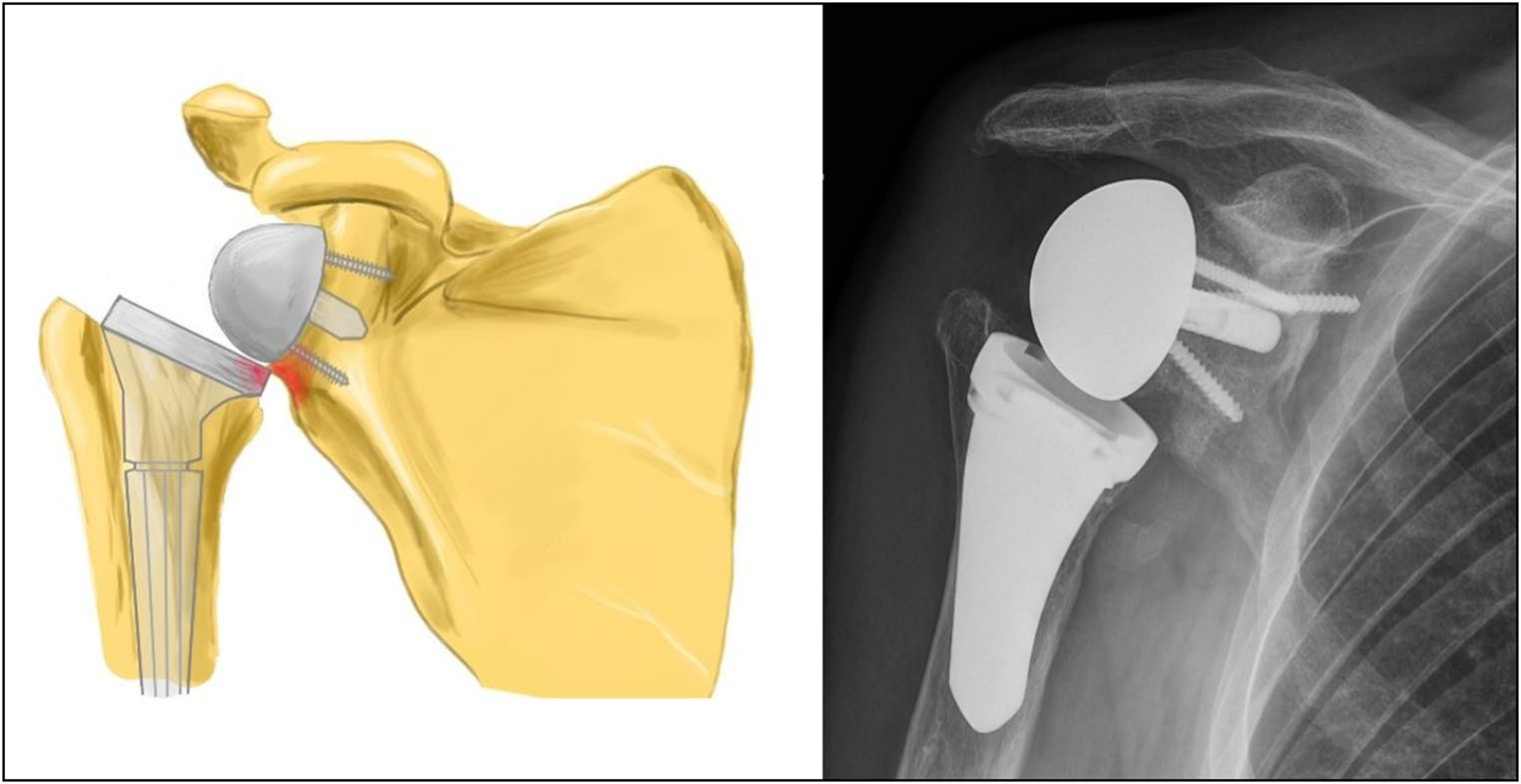
A Reverse Total Shoulder Arthroplasty (RTSA) in a patient with reduced Scapulo-humeral Angle (SHA) presenting scapular notching (same patient of Fig. 4). [Made with KRITA 4.4.8]

A diminished SHA results in a reduced distance between the scapular neck and the medial portion of the humeral prosthetic component in a RTSA (see Fig. 5).

The principal objective of this study was to evaluate the preoperative SHA and determine the presence and grade of SN at the latest available follow-up. The aim was to search for a correlation between these two parameters and gain insights into their relationship.

## Materials and methods

The authors structured this study in two distinct steps. In the initial phase, a comprehensive literature review was conducted in June 2022, utilizing PubMed/Medline, Cochrane, and Enbase databases. The objective was to identify all relevant studies pertaining to scapular notching and its associated risk factors. The databases were systematically queried using key terms such as “Scapular Notching,” “Reverse Total Shoulder Arthroplasty,” “Scapular Notching Risk Factors,” “Reverse Total Shoulder Arthroplasty Planning,” and “Reverse Total Shoulder Arthroplasty Complication.” This step was performed to identify and incorporate all potential risk factors associated with scapular notching (SN) into the study’s dataset.

The second step involved a retrospective examination of the hospital database, focusing on patients who underwent RTSA in the five-year period between January 1st, 2016, and December 31st, 2020. The inclusion and exclusion criteria outlined below were then applied to screen and select eligible patients.

### Inclusion criteria

Patients aged 18 years or older undergoing RTSA due to RCA.

Availability of a complete and standardized pre and post-operative X-ray protocol performed within the same orthopaedic institute.

### Exclusion criteria

Presence of open physis, fractures, tumors, local expanding lesions, foreign bodies, or signs of previous surgery.

Patients treated with RTSA for reasons other than RCA.

Incomplete pre or post-operative X-ray protocol.

The hospital’s imaging protocol included a two-view X-ray of the shoulder, featuring a true anteroposterior (AP) view (on the glenoid plane) with neutral external rotation and the arm adjacent to the patient’s body, as well as an axillary view with the arm in 45° of abduction.

Reports from a radiologist not involved in the study were consulted to verify the diagnosis of RCA. Only X-rays from the same hospital, performed following the hospital protocol, were subjected to analysis to minimize potential incongruities between radiographic study procedures.

The collected data included information on sex, age, affected side, preoperative SHA, presence of SN, SN grade according to the Nerot classification, RTSA model used, glenoid shape (eccentric or not), glenoid diameter, angle of the humeral neck component, preoperative scapular neck length, surgical approach, and follow-up duration.

The researchers employed the same image viewing program to analyze the exams and graded SN according to the Nerot classification into four grades. A safe cut-off for preoperative SHA was sought in terms of an increased risk of SN.

The study protocol received approval from the Regional Ethical Committee. All investigations were conducted at the same orthopaedic institute.

### Statistical analysis

The statistical analysis was conducted by one of the authors (A.M.) using Prism Version 6.0 software (GraphPad Software Inc) and RStudio Version 1.1.383 software (RStudio, Inc).

To assess the normal distribution of the sample, the Shapiro–Wilk normality test was employed. Continuous variables were presented as the mean ± standard deviation (SD) or medians with first and third quartiles [Q1–Q3]. Categorical variables were reported as the number of cases and frequencies, and differences were examined using the chi-squared test or Fisher’s exact test.

The correlation between preoperative Scapulo-Humeral Angle (SHA) and the presence and grade of Scapular Notching (SN) at the latest follow-up was explored using the Pearson coefficient (“r”) or Spearman coefficient (“ρ”) test, depending on the characteristics of the data distribution.

For all statistical analyses, the significance level was set at a P value < 0.05.

## Results

The authors initially screened a total of 213 patients who underwent RTSA. After applying exclusion criteria, 171 patients were deemed ineligible. Of these, 73 were excluded due to RTSA for acute proximal humeral fractures, 52 had signs of prior proximal humeral fractures or fixed dislocations on preoperative X-rays, three had locally expanding lesions, 16 showed radiological signs of previous surgery (mostly rotator cuff repair or Latarjet procedure), and 27 had incomplete radiological follow-up.

Forty-two patients met the eligibility criteria for this study, with a mean follow-up of 30.29 months (Range: 24–58 months). Demographic data are detailed in Table 1.

**Table 1:**
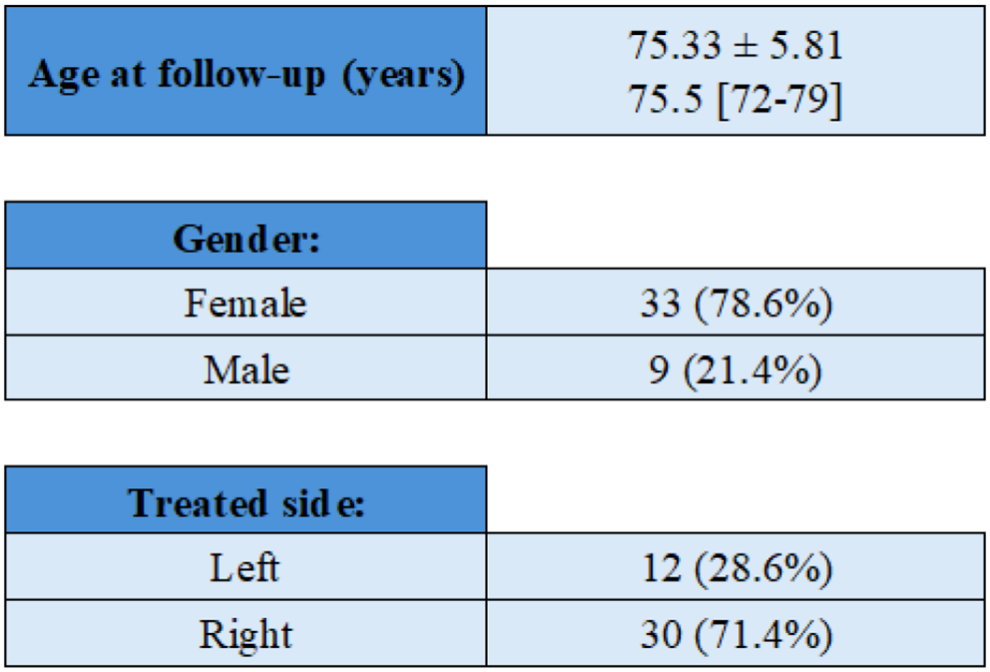
Patients’ demographics

*Data are reported as mean ± SD and median [Q1-Q3] or number of patients (percentage). Q1: first quartile; Q3: third quartile; SD: standard deviation*

Among the 42 patients, 12 exhibited some degree of SN, resulting in an incidence of 28.5%. The preoperative SHA had a mean value of 58° ± 21.4° (range: 22°-86°, 95% CI: 54.11°-61.84°).

Patients were treated with three different RTSA models (Table 2), all featuring an eccentric glenosphere.

**Table 2:**
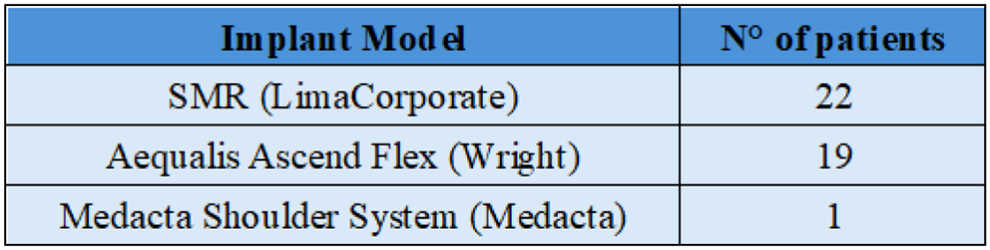
Implant Model

*Data are reported with the brand name of the model used. N° of patients: Number of Patients per type of implant*

Detailed information on implants and anatomical characteristics is presented in Table 3.

**Table 3:**
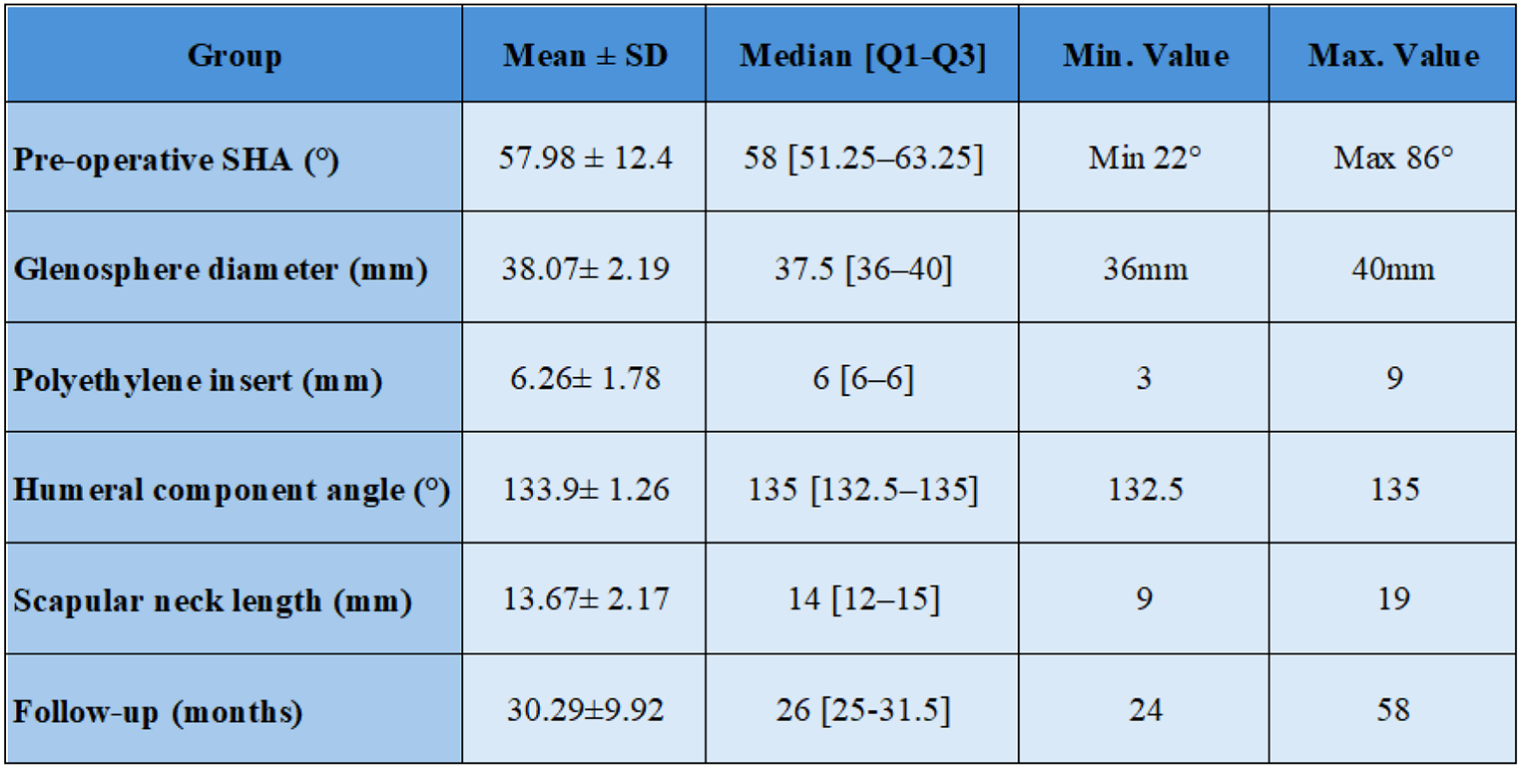
Summary of the main preoperative data and characteristics of the implant used

*Data are reported as mean ± SD and median [Q1-Q3] or number of patients (percentage). Q1: first quartile; Q3: third quartile; SD: standard deviation. Min: minimum value; Max: Maximum value*

Statistical analyses were conducted on all implants and anatomical characteristics to identify correlations with SN development. No significant differences were found in eccentric glenosphere, RTSA model, polyethylene insert dimension, glenosphere diameter, and humeral component angle regarding increased SN incidence.

A statistically significant Pearson coefficient correlation was observed between preoperative SHA and SN incidence (*r*= -0.6954; 95% confidence interval − 0.8250 to -0.4963; *p* < 0.0001). Similarly, a statistically significant Pearson coefficient correlation was found between the degree of SN (according to the Nerot classification) and preoperative SHA (*r*= -0.7045; 95% confidence interval − 0.8306 to -0.5096; *p* < 0.0001).

The only parameter, aside from preoperative SHA, showing statistical significance was a lower scapular neck length correlated with a higher SN incidence (*p* = 0.1021).

Among the 42 patients analyzed, 10 had a SHA lower than 50°, and all of them exhibited some degree of SN (100%). In contrast, out of the 32 patients with a SHA higher than 50°, only two presented grade 1 SN (6.25%).

## Discussion

The primary finding of this study reveals a statistically significant correlation between preoperative SHA and an increased incidence of post-operative SN. Additionally, the study demonstrates that a smaller preoperative SHA corresponds to a higher degree of SN.

Literature reports varying SN prevalence, ranging from 10.1% to as high as 75%.[11; 13; 21].

The overall prevalence of SN in this study was 28.5%, a figure that is challenging to compare directly with existing literature due to the specific inclusion criteria focusing on RTSA for RCA.

In the study, RTSA implants performed for fractures or inflammatory diseases were excluded. This decision was based on the hypothesis that the reduction in SHA is caused by chronic scapular dyskinesia, secondary to rotator cuff rupture, a condition that develops over several years. Patients with a history of prior surgery on the affected shoulder were also excluded due to the potential risk of iatrogenic arthropathy.

Recent research suggests a correlation between SN and poorer RTSA long-term clinical outcomes, with higher SN grades associated with an increased risk of aseptic glenoid loosening.[10; 18; 20].

The study recognizes already known preventive strategies for minimizing SN risk, such as the use of a more inferiorly centered glenosphere, lateralized RSA designs, the deltopectoral approach, and a humeral component with a lower neck-shaft angle as valid[2; 9; 12; 14; 16; 17; 21]. However, despite addressing these factors in surgical planning and execution, SN remains a common occurrence in shoulder surgery.

The study introduces a novel perspective by challenging the traditional notion of the scapula as a fixed bone. It posits that SN is not solely determined by fixed factors but involves a dynamic interplay within the scapulohumeral joint. The study suggests that in vivo, the scapula undergoes rotation during humeral abduction, contributing to a dynamic variability in the SHA. The compensatory upward scapular rotation observed in RCA might lead to chronic scapula-thoracic dyskinesia, resulting in a reduced SHA and predisposing patients to post-operative SN.

A strong, statistically significant association has been found between preoperative SHA and SN incidence, indicating that a smaller SHA not only increases the risk of SN but also leads to a more severe degree of SN. The study proposes a SHA cutoff of 50° as a potential safety indicator, dividing patients into high and low-risk categories for SN development. However, this cutoff requires validation through future studies.

The study acknowledges limitations, including its retrospective nature and the relatively small number of patients analyzed due to strict inclusion criteria. Despite these limitations, the findings emphasize the importance of considering dynamic scapular factors in understanding and addressing SN in RTSA for RCA cases.

This study positions itself as a pilot study, laying the groundwork for prospective investigations in the future. If the findings are validated, subsequent studies aimed at clinical applications will be pursued.

## Conclusion

In summary, the SHA emerges as a pivotal factor influencing the risk of developing SN in RTSA. A smaller SHA is consistently associated with a higher risk of SN, and the degree of severity of SN tends to increase as the SHA decreases. The authors have identified a SHA of 50° as a critical cutoff point, below which the risk of any degree of SN becomes notably elevated.

While acknowledging the importance of addressing other recognized risk factors for SN in surgical planning, the study underscores that these factors may not fully compensate for an extremely low SHA. The findings emphasize the need for surgeons to consider SHA as a crucial dynamic element in the scapulohumeral joint during RTSA procedures.

The clinical implications of these findings are significant but warrant further exploration through prospective studies.

## Data Availability

The data presented in this study are available on request from the corresponding author. The data are not publicly available due to privacy and ethical restrictions.
